# Radiological Spectrum of Leptomeningeal Medulloblastoma: A Case-Based Literature Review With an Additional Case Report

**DOI:** 10.7759/cureus.101767

**Published:** 2026-01-18

**Authors:** Elif Cigdem Karatayli, Sükriye Yilmaz, Hasan Bulut, Efe Yetisgin, Sule Yesil, Muhammed Erkan Emrahoğlu

**Affiliations:** 1 Radiology, Ankara Etlik City Hospital, Ankara, TUR; 2 Pediatric Radiology, Ankara Etlik City Hospital, Ankara, TUR; 3 Pathology, Ankara Etlik City Hospital, Ankara, TUR; 4 Pediatric Oncology, Ankara Etlik City Hospital, Ankara, TUR; 5 Neurosurgery, Ankara Etlik City Hospital, Ankara, TUR

**Keywords:** brain tumors, cns tumors, leptomeningeal carcinoma, leptomeningeal enhancement, medulloblastoma, primary leptomeningeal medulloblastoma

## Abstract

Medulloblastomas are the most common malignant brain tumors in pediatric patients, typically arising from the cerebellar vermis within the posterior fossa. These neoplasms belong to the group of small round blue cell tumors and can be subdivided based on molecular profiling. While leptomeningeal spread is well recognized in advanced disease, primary leptomeningeal medulloblastoma (PLMB) without an identifiable intracranial mass is exceptionally uncommon and poses a diagnostic challenge due to overlap with infectious, inflammatory, and other neoplastic leptomeningeal conditions. Against this background, the recognition of atypical clinical presentations and subtle imaging findings is critical.

In this setting, we report a 16-year-old boy who presented with progressive lower-extremity weakness. Brain magnetic resonance imaging (MRI) demonstrated cerebellar and parahippocampal diffusion restriction with minimal leptomeningeal enhancement and no discrete parenchymal mass. Spinal MRI revealed diffuse intradural-extramedullary nodules with widespread leptomeningeal involvement. Histopathology confirmed a desmoplastic/nodular medulloblastoma, non-wingless (WNT)/non-sonic hedgehog (SHH).

When considered alongside the published literature, this case reflects the substantial variability in imaging findings, including inconsistent leptomeningeal enhancement, occasional diffusion restriction, and a high frequency of spinal metastases at presentation. Notably, clinical signs of intracranial hypertension, often anticipated in leptomeningeal disease, may be absent, further complicating timely diagnosis. By presenting this additional pediatric case and synthesizing current evidence, this report aims to refine the understanding of the radiological spectrum of non-mass-forming medulloblastoma and highlight the importance of recognizing subtle neuroaxis abnormalities suggestive of this rare entity.

## Introduction

Medulloblastomas are the most common pediatric malignant brain tumors. They are often located in the midline of the posterior fossa. Most medulloblastomas arise from the cerebellar vermis and protrude into the fourth ventricle. Drop metastases via cerebrospinal fluid (CSF) and leptomeningeal metastases are common pathways of spread [1-3]. Clinically, presentation is most often characterized by signs of intracranial hypertension. Additional manifestations may include cerebellar symptoms or neurological deficits secondary to spinal axis involvement [4,5].

Although leptomeningeal dissemination from mass-forming medulloblastomas is commonly encountered, primary leptomeningeal medulloblastoma (PLMB) without an identifiable mass remains exceedingly rare [4,5]. In this context, we present a case-based literature review including 20 previously reported cases, exploring the radiological spectrum of this rare entity and anchored by a representative case of a 16-year-old boy with primary leptomeningeal medulloblastoma and spinal drop metastases.

## Case presentation

A previously healthy 16-year-old boy presented to our institution with leg pain and progressive left-sided lower-extremity weakness, most evident during ambulation and stair climbing, accompanied by foot drop and gait instability. Over time, the weakness progressed to involve both lower extremities, resulting in bilateral foot drop and eventual inability to ambulate without support. Neurological examination demonstrated both proximal and distal weakness, without features suggestive of a focal lower motor neuron lesion. Sensory assessment revealed bilateral sensory impairment, initially more prominent on the left side, with a gradual reduction in proprioception, most evident at the toes and ankles, while relative preservation was noted at the knee. Taken together, the distribution and progression of the motor and sensory deficits favored a central neurological pathology rather than an isolated peripheral nerve disorder. No other abnormalities were noted on physical examination, and the laboratory workup was unremarkable.

**Figure 1:**
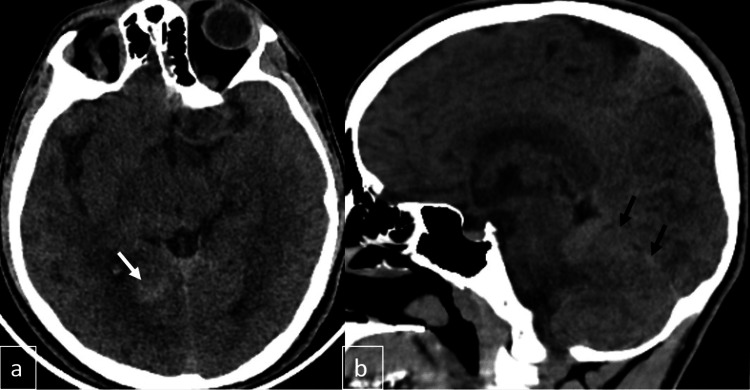
Axial CT image (a) shows a poorly defined hyperdense area in the right posterior parahippocampal region, while sagittal CT image (b) demonstrates mild cerebellar parenchymal hyperdensity with folial effacement suggestive of edema. CT: computed tomography

A computed tomography (CT) scan was performed to rule out any acute central pathologies. No significant intracranial hemorrhage was detected on the scan. A small hyperdense area with ill-defined borders was noted in the right posterior parahippocampal region, demonstrating asymmetry relative to the contralateral side. Mild edema was observed in the cerebellar parenchyma, along with a white cerebellum-like appearance (Figure 1). To facilitate a comprehensive evaluation of the identified findings and to assess the potential for a neoplastic or inflammatory process, the patient underwent a magnetic resonance imaging (MRI) of the brain and the entire spine, including the cervical, thoracic, and lumbar regions.

**Figure 2:**
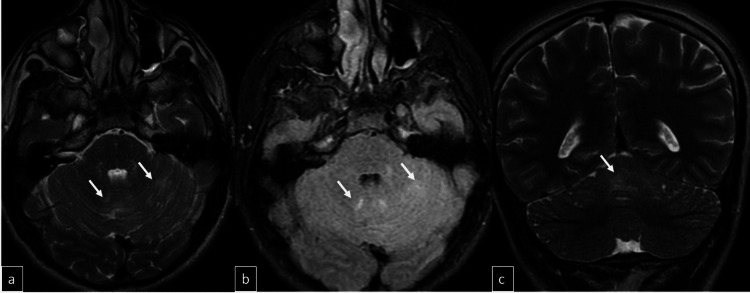
Patchy areas of increased signal intensity with folial effacement consistent with edema are observed in the cerebellum on axial T2-weighted (a), FLAIR (b), and coronal T2-weighted (c) images (arrows). FLAIR: fluid-attenuated inversion recovery

**Figure 3:**
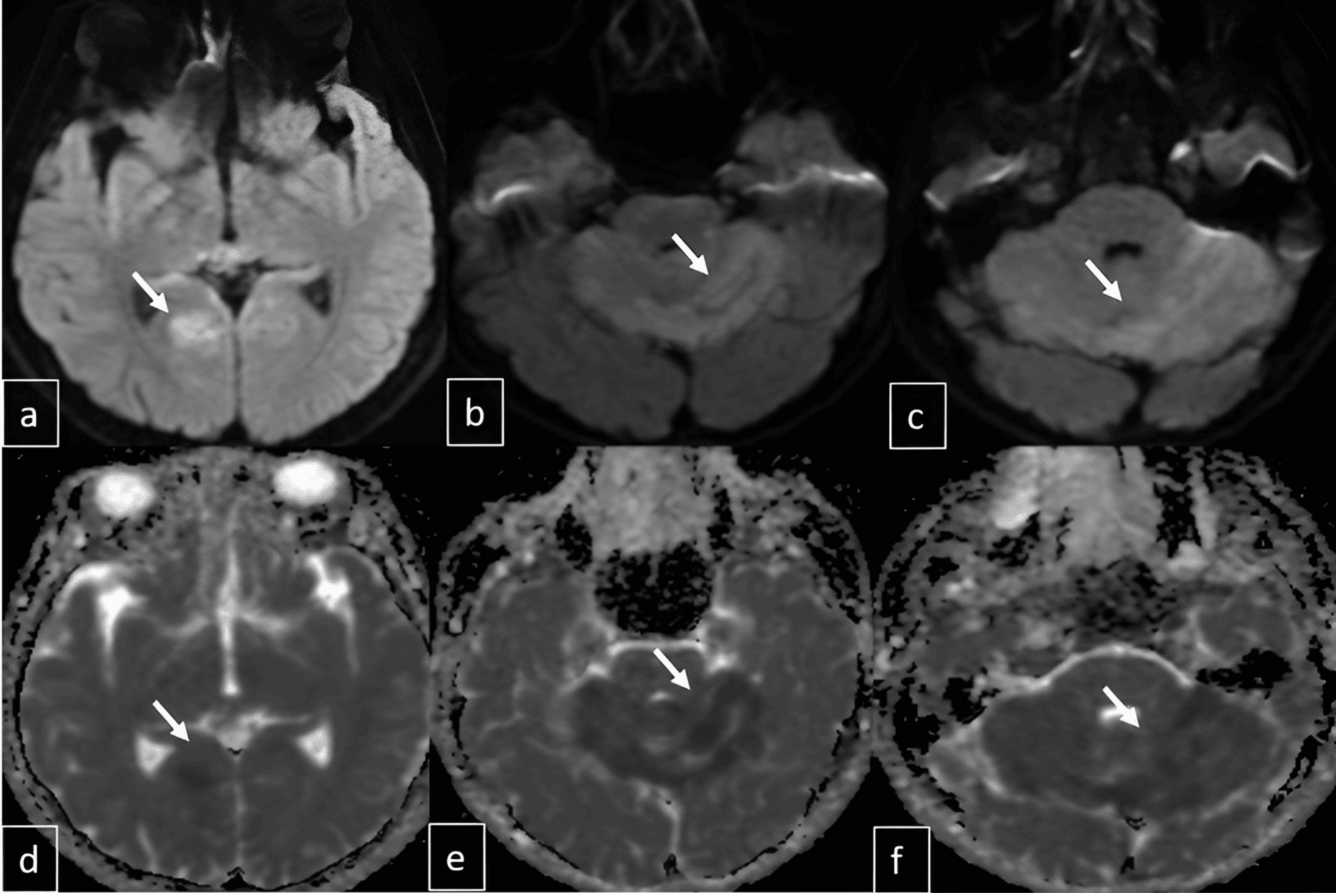
Diffusion restriction is noted in the right posterior parahippocampal region and cerebellar parenchyma on diffusion-weighted images (a-c) and apparent diffusion coefficient maps (d-f), as indicated by the arrows.

**Figure 4:**
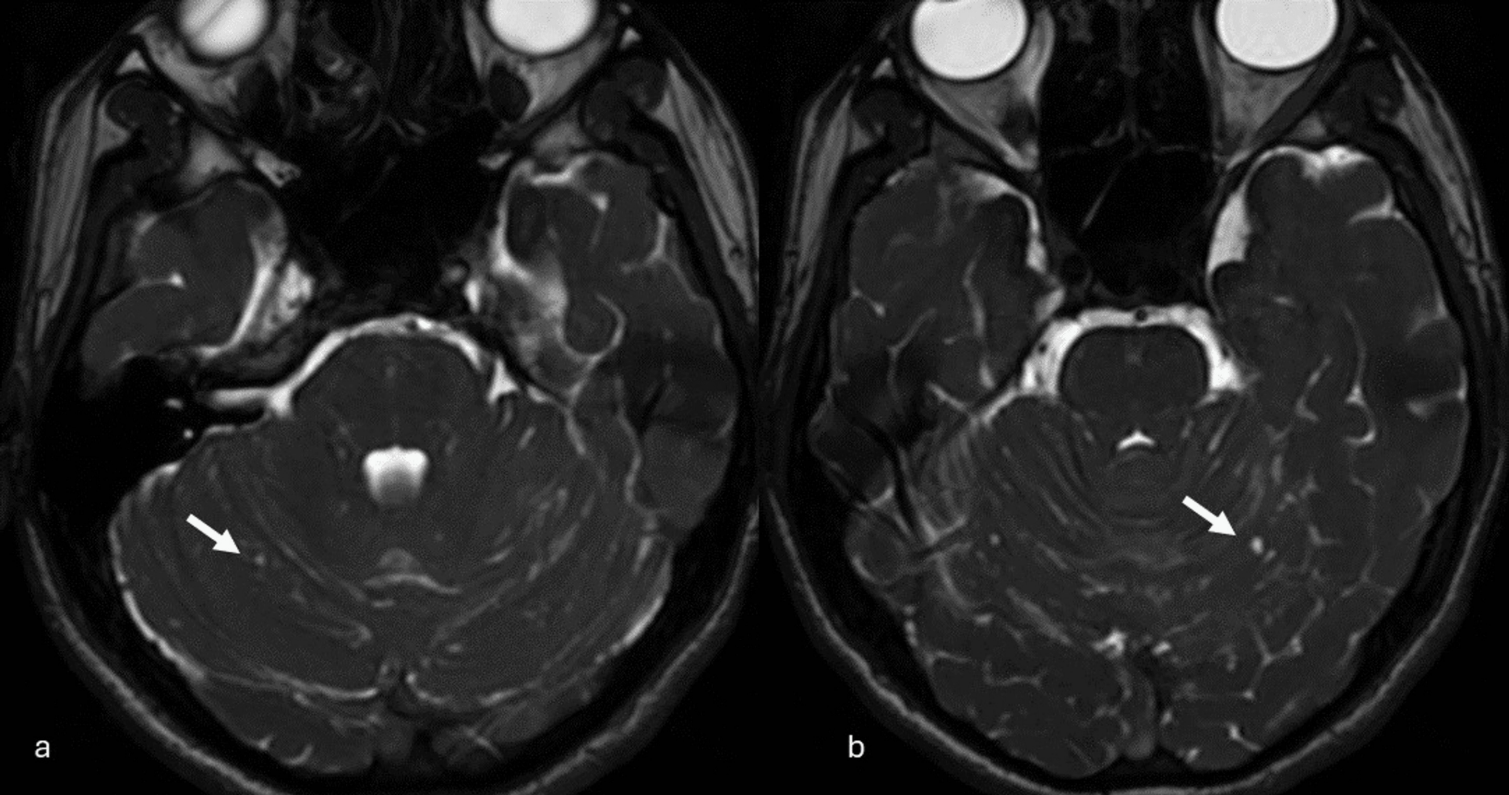
Millimetric cysts are observed in the cerebellar parenchyma on axial FIESTA images (a and b), indicated by the arrows. FIESTA: Fast Imaging Employing Steady-State Acquisition

**Figure 5:**
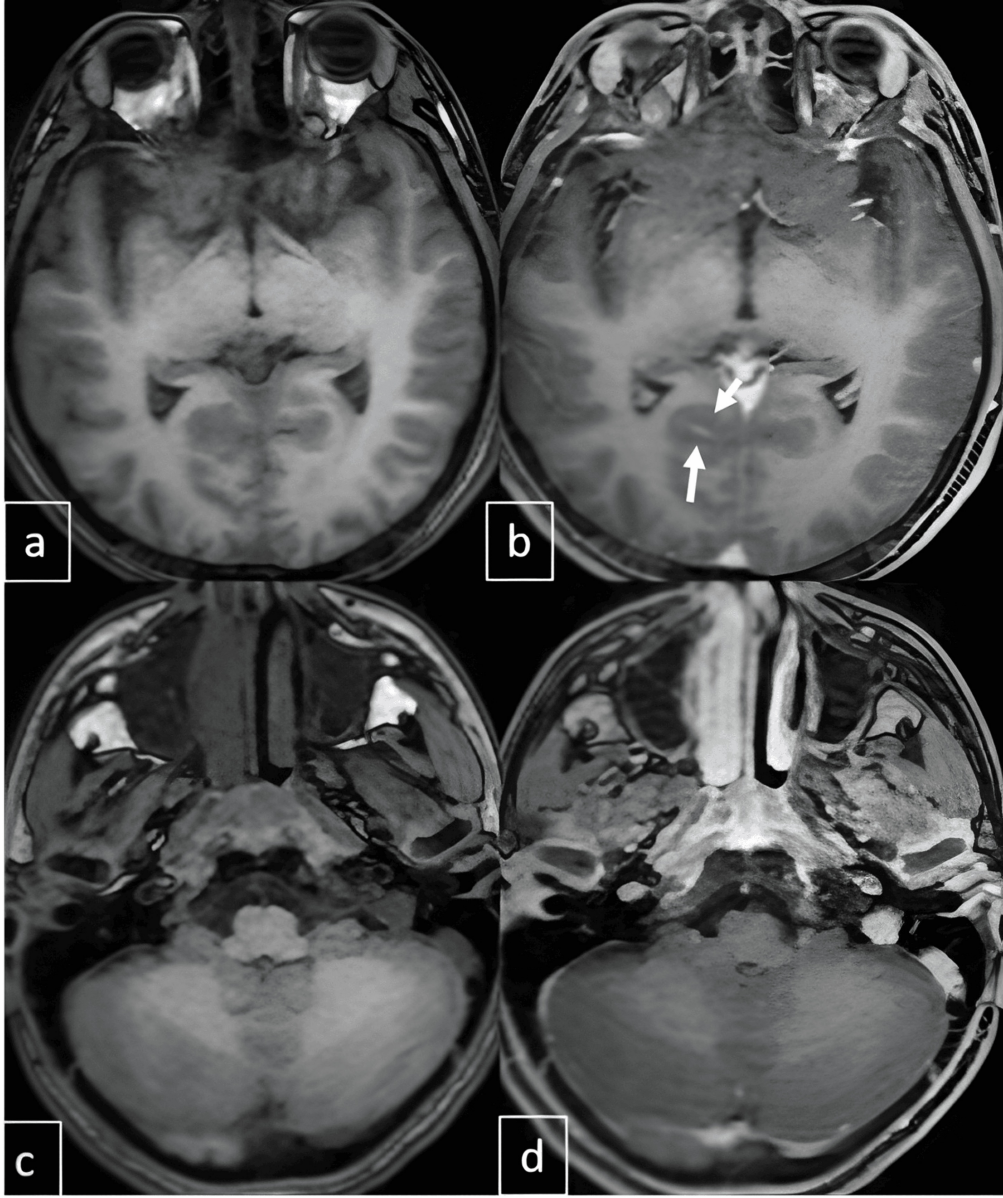
On axial pre-contrast (a and c) and post-contrast (b and d) T1-weighted images, no significant enhancement is observed within the cerebellar parenchyma; however, leptomeningeal enhancement is evident in the right posterior parahippocampal region, as indicated by the arrow (b).

**Figure 6:**
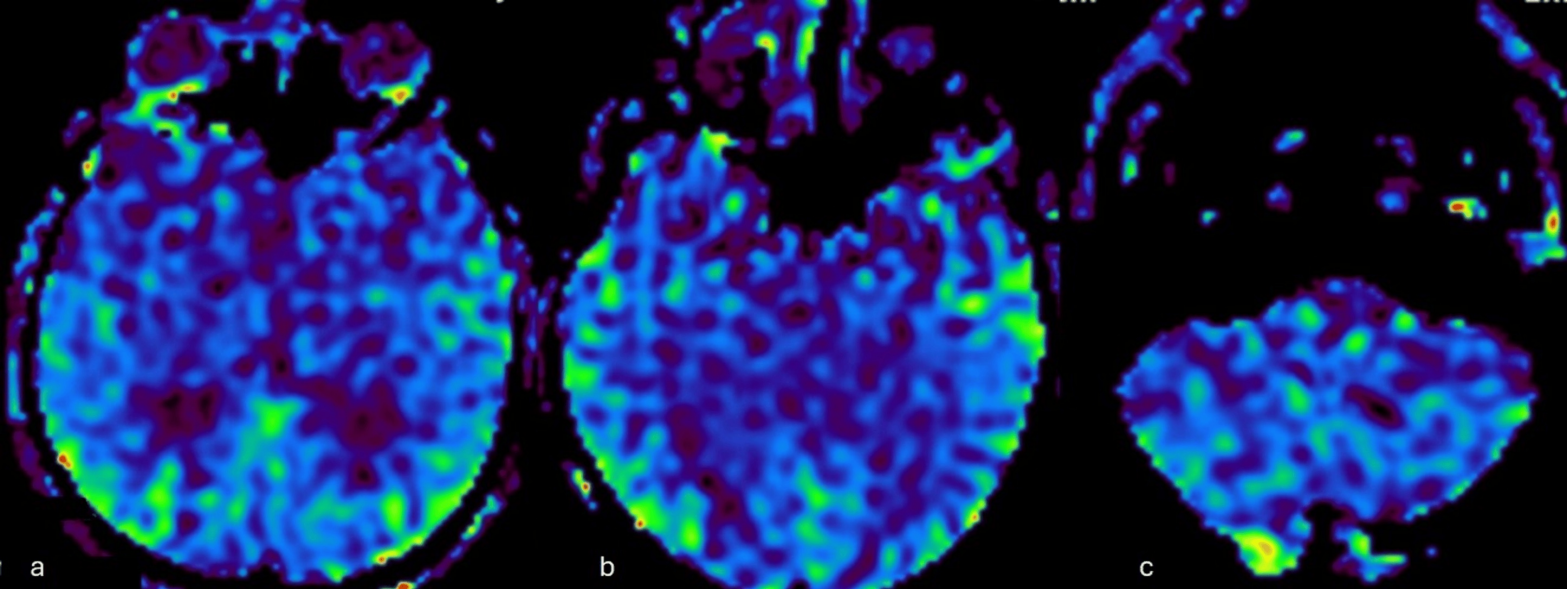
Arterial spin labeling perfusion images (a-c) show no significant perfusion difference or increase in cerebral blood flow.

Subsequent cranial MRI (1.5 Tesla GE Signa Explorer, GE HealthCare, Chicago, IL) demonstrated mildly hyperintense areas on T2-weighted and fluid-attenuated inversion recovery (FLAIR) sequences, with marked diffusion restriction in both cerebellar hemispheres, the vermis, tectal surfaces, and posterior parahippocampal regions (Figures 2, 3). On the axial Fast Imaging Employing Steady-State Acquisition (FIESTA) (GE HealthCare) sequence, millimetric cystic structures were identified within the cerebellar parenchyma; however, their precise localization, whether intraparenchymal or within the leptomeningeal space, could not be definitively determined (Figure 4). While very subtle leptomeningeal contrast enhancement was noted in the prominent right posterior parahippocampal region, no significant contrast enhancement was observed in the cerebellar region (Figure 5). Contrast-enhanced FLAIR sequences were not performed, as they are not routinely acquired in our clinic. The arterial spin labeling (ASL) perfusion examination revealed no areas of significantly increased perfusion or notable perfusion asymmetries within the brain parenchyma (Figure 6). However, no discrete mass formation was identified.

**Figure 7:**
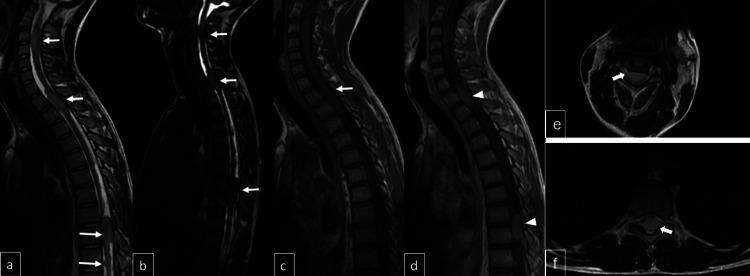
Sagittal T2-weighted (a), FIESTA (b), T1-weighted (c), and post-contrast T1-weighted (d) images demonstrate intradural nodular lesions within the spinal canal (arrows) with contrast enhancement (arrowheads), while axial T2-weighted images (e and f) confirm their intradural extramedullary localization (arrows). FIESTA: Fast Imaging Employing Steady-State Acquisition

An MRI of the cervical, thoracic, and lumbar spine demonstrated multiple intradural-extramedullary masses within the spinal canal, with the largest lesion located at the T8-T9 level, resulting in the significant compression of the spinal cord. The identified spinal lesions caused significant spinal canal narrowing, leading to substantial spinal cord compression, with evidence of focal cord invasion in certain regions. Additionally, diffuse leptomeningeal contrast enhancement with nodular morphology was observed along the entire spinal cord, consistent with leptomeningeal metastases (Figure 7).

Given the patient's progressive lower-extremity weakness and compressive symptoms, decompressive surgery was performed to relieve spinal cord compression. This included multilevel decompression with laminectomy and tumor biopsy.

**Figure 8:**
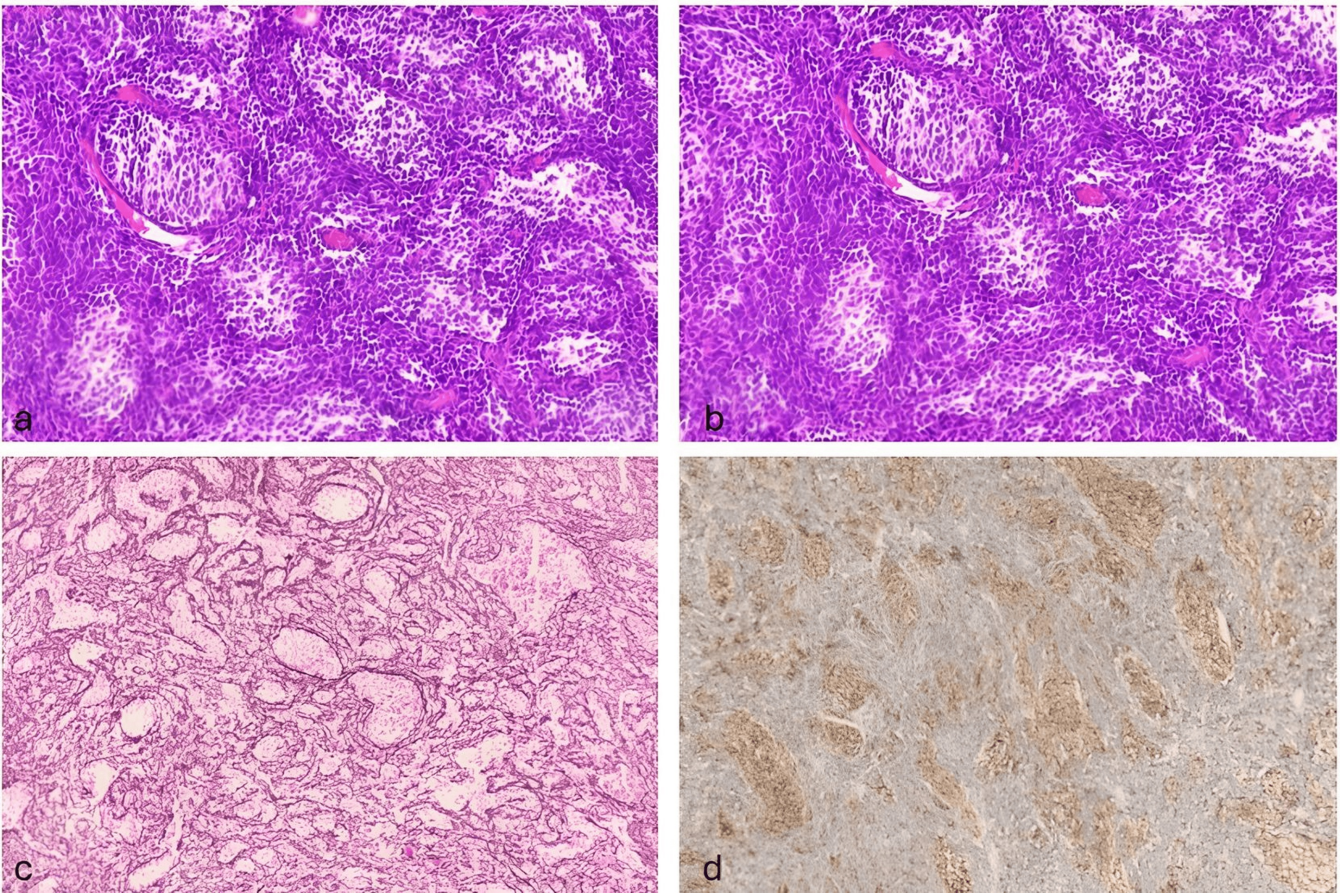
H&E staining at 200× (a and b) shows pale nodules surrounded by undifferentiated cells with hyperchromatic, pleomorphic nuclei; reticulin staining (c) highlights a network encasing the pale nodules, while synaptophysin staining (d) demonstrates diffuse cytoplasmic positivity in tumor cells.

Following histopathological analysis, the patient was diagnosed with medulloblastoma, desmoplastic/nodular variant, classified as non-wingless (WNT)/non-sonic hedgehog (SHH) according to the molecular classification system outlined in the WHO 2021 classification (Figure 8).

## Discussion

Medulloblastomas usually present in childhood, with two peak incidences at ages 1-4 and 5-9 years. There is a moderate male predilection. Although rare, medulloblastomas can occur in adults, typically in the third and fourth decades of life. Patients frequently present with headaches and signs of increased intracranial pressure secondary to obstructive hydrocephalus [1-3].

Medulloblastoma is one of the small round blue cell tumors and is highly cellular. In the fifth edition of the WHO classification of CNS tumors (2021), medulloblastomas are classified as embryonal, grade 4 tumors and are molecularly divided into four subgroups based on DNA methylation and/or transcriptome profiling (Table 1). Due to the high incidence of metastases at presentation, early and comprehensive neuroaxis screening is essential for accurate staging and treatment planning [1-3].

**Table 1:** The molecular subgroups of medulloblastoma and characteristically altered genes. Source: [1]

Medulloblastomas, Molecularly Defined	Genetic and Molecular Alterations
Wingless (WNT)-activated	CTNNB1 and APC
Sonic hedgehog (SHH)-activated and TP53-wildtype	TP53, PTCH1, SUFU, SMO, MYCN, and GLI2 (methylome)
Sonic hedgehog (SHH)-activated and TP53-mutant	
Non-WNT/non-SHH	MYC, MYCN, PRDM6, and KDM6A (methylome)

The densely packed cellular structure of the tumor is reflected in its radiological features. On CT, most medulloblastomas appear hyperdense on non-contrast scans and typically demonstrate contrast enhancement. Necrosis is not uncommon, and calcifications may be present [2,3,6]. On MRI, medulloblastomas appear iso- to hyperintense to gray matter on T2-weighted images and exhibit diffusion restriction. They often show heterogeneous contrast enhancement. Magnetic resonance (MR) spectroscopy reveals elevated choline and decreased N-acetylaspartate (NAA), similar to other malignant tumors. Taurine, which resonates at 3.4 parts per million (ppm), is also elevated [2,3,7]. Recent literature suggests that tumor location may predict molecular subgroups. Tumors originating from the cerebellopontine angle or cerebellar peduncles are most likely WNT-activated tumors and have the best prognosis. Tumors originating from the cerebellar hemisphere are most likely to belong to the SHH subgroup. Tumors located in the midline may belong to non-WNT/non-SHH (group 3 or 4) [1,8].

Primary leptomeningeal medulloblastoma (PLMB) without mass formation is extremely rare. The existing literature documents a limited number of cases; including the present case, 21 cases of primary leptomeningeal medulloblastoma have been reported across a wide age range (Table 2). PLMB predominantly affects pediatric patients (57%) with a slight male predominance. Headache and symptoms related to intracranial hypertension were the most frequent presenting features. Upon admission, our patient did not demonstrate signs of hydrocephalus or elevated intracranial pressure; however, spinal drop metastases and clinical findings indicative of spinal cord compression, including extremity-related symptoms, were noted [4,5,9-25].

**Table 2:** Reported cases of primary leptomeningeal medulloblastoma, including the present case: clinical, imaging, and molecular characteristics. *Primary spinal leptomeningeal medulloblastoma. MRI, magnetic resonance imaging; CT, computed tomography; NA, not available; DWI, diffusion-weighted imaging; FLAIR, fluid-attenuated inversion recovery; SHH, sonic hedgehog; WNT, wingless; WT, wildtype

Number	Reference	Age	Sex	Clinical Presentation	Imaging Modality	Leptomeningeal Enhancement	DWI Restriction	Posterior Fossa T2/FLAIR	CT Hyperdensity	Hydrocephalus	Spinal Involvement	Other Imaging Findings	Molecular/Genetic
1	Russo et al., 2022 [4]	18 months	Male	Fever, seizures, and rapid deterioration of consciousness	MRI	Present	Present	NA	NA	Present	Present	Absent	non-SHH/non-WNT
2	McGlacken-Byrne et al., 2018 [9]	2 years	Male	Vomiting, lethargy, irritability, headache, and ataxia	MRI	Present	Absent	Present	NA	Absent	Present	Absent	non-SHH/non-WNT
3	Russo et al., 2022 [4]	3 years	Male	Irritability, frontal headache, and emesis	MRI and CT	Present	Present	NA	NA	Present	Present	Absent	non-SHH/non-WNT
4	Vallejo Díaz et al., 2023 [10]	4 years	Male	Persistent headache, nocturnal vomiting, weight loss, and ataxia	MRI and CT	Present	Present	Present	Absent	Present	Present	Absent	MYC amplification
5	Ferrara et al., 1989 [11]	5 years	Male	Intracranial hypertension	CT	Present	NA	NA	Absent	Absent	Absent	Absent	NA
6	Mehta et al., 2009 [12]	8 years	Male	Headache and mild visual impairment	MRI	Present	Absent	Absent	Absent	Absent	Absent	Absent	NA
7	Suman et al., 2007 [13]	10 years	Female	Progressive headache and vomiting	MRI	Present	NA	Absent	NA	Absent	Present	Absent	NA
8	Morgacheva et al., 2022 [5]	11 years	Female	Fatigue, fever, headache, vomiting, diplopia, and visual loss	MRI	Present	NA	NA	NA	Present	Present	Periventricular edema	WNT
9	Ghosh et al., 2018 [14]	11 years	Male	Headache, seizure, and vomiting	MRI and CT	Present	NA	NA	NA	Present	Absent	Absent	non-SHH/non-WNT
10	Meister et al., 2022 [15]	14 years	Male	Diplopia, headache, facial numbness, and gait disturbance	MRI	Present	NA	NA	NA	Present	Present	Absent	non-SHH/non-WNT
11	Kajtazi et al., 2021 [16]	18 years	Female	Leg weakness, visual blurring, and headache	MRI	Present (delayed)	NA	NA	NA	Absent	Present (primary)	Optic nerve sheath thickening, optic disk edema, and dural sinus stenosis	*
12	Noiphithak et al., 2021 [17]	19 years	Male	Chronic suboccipital headache	MRI	Present (delayed)	Absent	Absent	Absent	Absent	Present	Tonsillar descent and optic disk edema	Group 4
13	Guo et al., 2012 [18]	21 years	Male	Headache, diplopia, and tinnitus	MRI	Present	Absent	Absent	NA	Present	Present	Delayed nodular enhancement	NA
14	Asadollahi et al., 2012 [19]	22 years	Male	Headache, hearing loss, and blurred vision	MRI	Present	Absent	Present	NA	Absent	NA	Absent	NA
15	Barlas et al., 2021 [20]	24 years	Female	Headache and lower-extremity weakness	MRI	Present	NA	NA	NA	Absent	Present	Absent	YAP1+ and GAD-
16	Rushing et al., 2009 [21]	30 years	Male	Neck pain	MRI and CT (cervical spinal)	NA	Absent	Absent (perceived in second look)	Absent	Absent	Present	Tonsillar descent	NA
17	Ala et al., 2020 [22]	34 years	Male	Diplopia, headache, and blurred vision	MRI	Absent	Present	Present	Absent	Absent	Present	Optic nerve sheath thickening, optic disk edema, and dural sinus stenosis	SHH and TP53-WT
18	Hey et al., 2025 [23]	34 years	Female	Acute bilateral radiculopathy	MRI	Present	Absent	Absent	NA	Absent	Present	Syrinx	SHH and TP53-WT
19	Fabbro et al., 2022 [24]	35 years	Female	Headache, diplopia, and ataxia	MRI	Absent	NA	Present	NA	Present	Present	Tonsillar descent	NA
20	Hankey et al., 1989 [25]	39 years	Female	Headache, ataxia, and hallucinations	CT	Present (delayed)	NA	NA	Absent	Absent	Absent	Absent	NA
21	Our case	16 years	Male	Lower-extremity weakness with radiculopathy	MRI and CT	Absent	Present	Present	Present	Absent	Present	Absent	non-SHH/non-WNT

Radiologically, the predominant imaging pattern consists of leptomeningeal contrast enhancement, most commonly involving the posterior fossa and characteristically occurring in the absence of a discrete parenchymal mass lesion. Leptomeningeal enhancement was observed at some point during the disease course in 95% of cases, although it was absent at initial imaging in nearly one-quarter. Additional imaging findings have been variably reported, including diffusion restriction, posterior fossa T2/FLAIR abnormalities, hydrocephalus, and imaging features of intracranial hypertension, likely related to the obstruction of cerebrospinal fluid pathways [4,5,9-25].

Spinal involvement was common, occurring in roughly two-thirds of cases, with rare instances of primary spinal disease. Other described imaging findings include tonsillar descent or herniation, delayed cerebellar parenchymal lesions, and syrinx formation. Consistent with these reports, our patient demonstrated nodular spinal cord metastases, without an initial dominant cerebellar parenchymal mass [4,5,9-25].

Molecular profiling, when available, revealed heterogeneous subtypes, most frequently within non-WNT/non-SHH categories, whereas molecular data were unavailable in a substantial proportion of cases [4,5,9-25].

The main differential diagnoses of primary leptomeningeal medulloblastoma include acute cerebellitis, meningitis, diffuse leptomeningeal glioneuronal tumor, leptomeningeal carcinomatosis from other primaries, and, particularly in young adult patients, Lhermitte-Duclos disease. Despite the presence of characteristic imaging and clinical findings in these conditions, definitive diagnosis can be established by biopsy.

Patients with acute cerebellitis or meningitis typically present with infectious symptoms, and their imaging findings may be indistinguishable from those of primary leptomeningeal medulloblastoma. In addition, immune-mediated conditions such as acute disseminated encephalomyelitis (ADEM) can further complicate the differential diagnosis. Therefore, in patients who fail to respond to appropriate antibiotic or corticosteroid therapy, leptomeningeal malignancy should be considered [9,26].

Diffuse leptomeningeal glioneuronal tumor is another rare tumor that presents as leptomeningeal enhancement without a mass. On MRI, small subpial cysts that appear hyperintense on T2 and FLAIR and hypointense on T1 are helpful in diagnosis [27]. A review of the literature reveals no documented instances of leptomeningeal medulloblastoma presenting with small cerebellar cystic formations. The presence of these cysts in our patient highly prompted the consideration of diffuse leptomeningeal glioneuronal tumor as part of the differential diagnosis.

Lhermitte-Duclos disease, also known as dysplastic cerebellar gangliocytoma, typically affects one hemisphere. Widened and T2 hyperintense cerebellar folia create a characteristic "tigroid" pattern on MRI. These lesions do not exhibit diffusion restriction, and contrast enhancement is rare [28].

The prognosis of primary leptomeningeal medulloblastoma is poorer than that of conventional medulloblastomas, and surgical intervention is usually not feasible; moreover, reported survival is extremely limited, with several patients surviving only weeks to months after diagnosis [4,5,9-25].

The absence of contrast-enhanced FLAIR imaging, which is not routinely performed in our institution, represents a limitation in our case, as recent literature suggests that this sequence might improve the detection of leptomeningeal disease [29].

## Conclusions

Primary leptomeningeal medulloblastoma is a rare entity, and its diagnosis is challenging because of significant overlap with infectious, inflammatory, and other neoplastic leptomeningeal diseases. The awareness of the differential diagnosis in leptomeningeal disorders is essential, particularly when imaging demonstrates diffuse leptomeningeal enhancement in the absence of a discrete parenchymal mass, to facilitate early recognition and appropriate management.

We present this case to emphasize specific manifestations and to highlight key radiological features of the disease, with the intent of improving diagnostic acumen and facilitating prompt recognition in subsequent cases.
